# Comparison of Different Approaches to Operationalize Fried’s Phenotypic Frailty in the NuAge Cohort

**DOI:** 10.1093/geroni/igab046.1479

**Published:** 2021-12-17

**Authors:** Valerie Turcot, Alan Cohen, Pierrette Gaudreau, Véronique Legault, José Morais, Nancy Presse, Stéphanie Chevalier

**Affiliations:** 1 CIUSSS de l’Estrie-CHUS, Sherbrooke, Quebec, Canada; 2 Universite de Sherbrooke, Sherbrooke, Quebec, Canada; 3 Université de Montréal, Montreal, Quebec, Canada; 4 Université de Sherbrooke, Sherbrooke, Quebec, Canada; 5 McGill University, Montreal, Quebec, Canada; 6 McGill University, Ste-Anne-de-Bellevue, Quebec, Canada

## Abstract

Many operationalization approaches were proposed to identify frailty in older adults. The common use of Fried’s original criteria or other cut-offs based on cohort distribution may not apply in every cohort leading to potential bias in the identification of frail individuals. We thus aimed to apply different Fried’s phenotypic frailty operationalization approaches in the Quebec NuAge cohort of generally healthy community-dwelling older adults (n=1,753; aged 67-84 years), and longitudinally compare prevalence, incidence and predictive strength on outcomes, such as functional autonomy, falls, hospitalization and mortality. Significant variability in prevalence, classification agreement and predictive strengths were observed between approaches, notably using different types of distribution cut-offs, variables, or ways to handle missing data. This strategy helped us to prioritize a specific Fried’s phenotypic frailty operationalization in NuAge, which could then be used in secondary research projects aiming to study determinants of Fried’s phenotypic frailty and its role in health outcomes.

